# Supplementary material for: Predominance of BRCA2 Mutation and Estrogen Receptor Positivity in Unselected Breast Cancer with BRCA1 or BRCA2 Mutation
Source: Cancers (Basel). 2022 Jul 4;14(13):3266. doi: 10.3390/cancers14133266 (PMC9265086; doi:10.3390/cancers14133266)
Supplement: Supplementary file 1 [file cancers-14-03266-s001.zip › cancers-1754139-supplementary.pdf]

Supplemental data

Summary

Figure S1. Subgroup analysis of progression free survival in ER+ gBRCAbreast cancer patients treated with PARPi in phase III trial 2

Figure S2. Quoron flowchart 3

Table S1: Frequency of gBRCA1 and gBRCA2 carriers in unselected breast cancer 3

Table S2. Frequency of gBRCA1 and gBRCA2 carriers by ER status in unselected breast cancer 6

Table S3. Frequency of gBRCA carriers in unaffected individuals 6

Table S4. Frequency of gBRCA carriers in gnomAD* exome sequencing database** 7

Table S5. Meta-analysis of gBRCA carriers in unselected breast cancer 2

Table S6. Meta-analysis of gBRCA carriers in ER+ breast cancer 2

Table S7. Meta-analysis of gBRCA in ER- breast cancer 10

Table S8. Meta-analysis of gBRCAin unaffected individuals 12

Table S9. Meta-analysis of gBRCA1 carriers in unselected breast cancer 13

Table S10. Meta-analysis of gBRCA2 carriers in unselected breast cancer 14

Table S11. Meta-analysis of gBRCA1 carriers among unaffected individuals ……………………..14

Table S12. Meta-analysis of gBRCA2 carriers in unaffected individuals 16

Figure S1. Subgroup analysis of progression-free survival in ER+ gBRCA breast cancer treated with a PARPi in a phase III trial

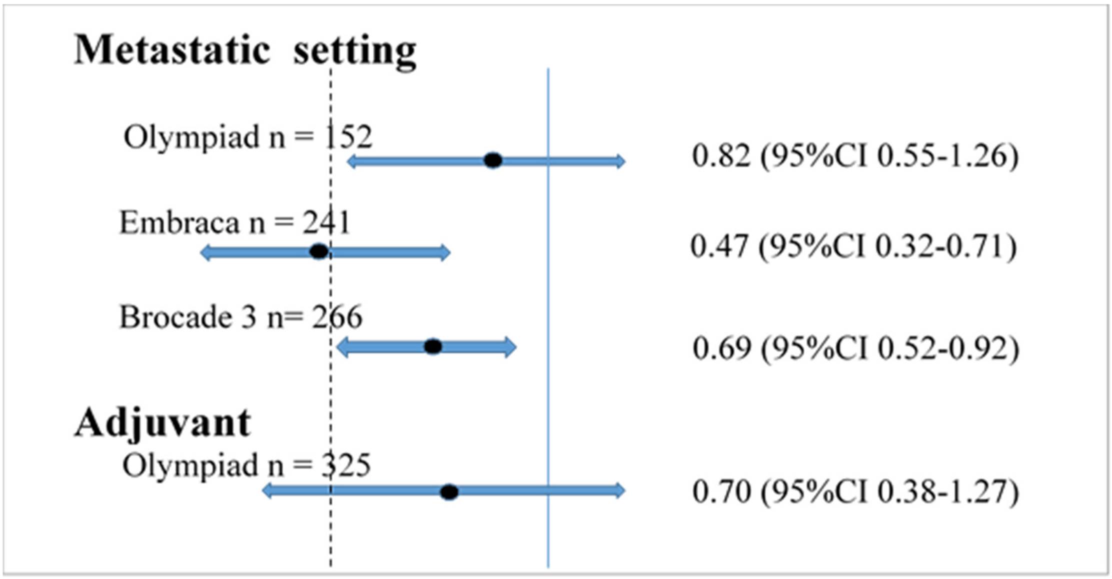

Figure S2. Quoron flowchart

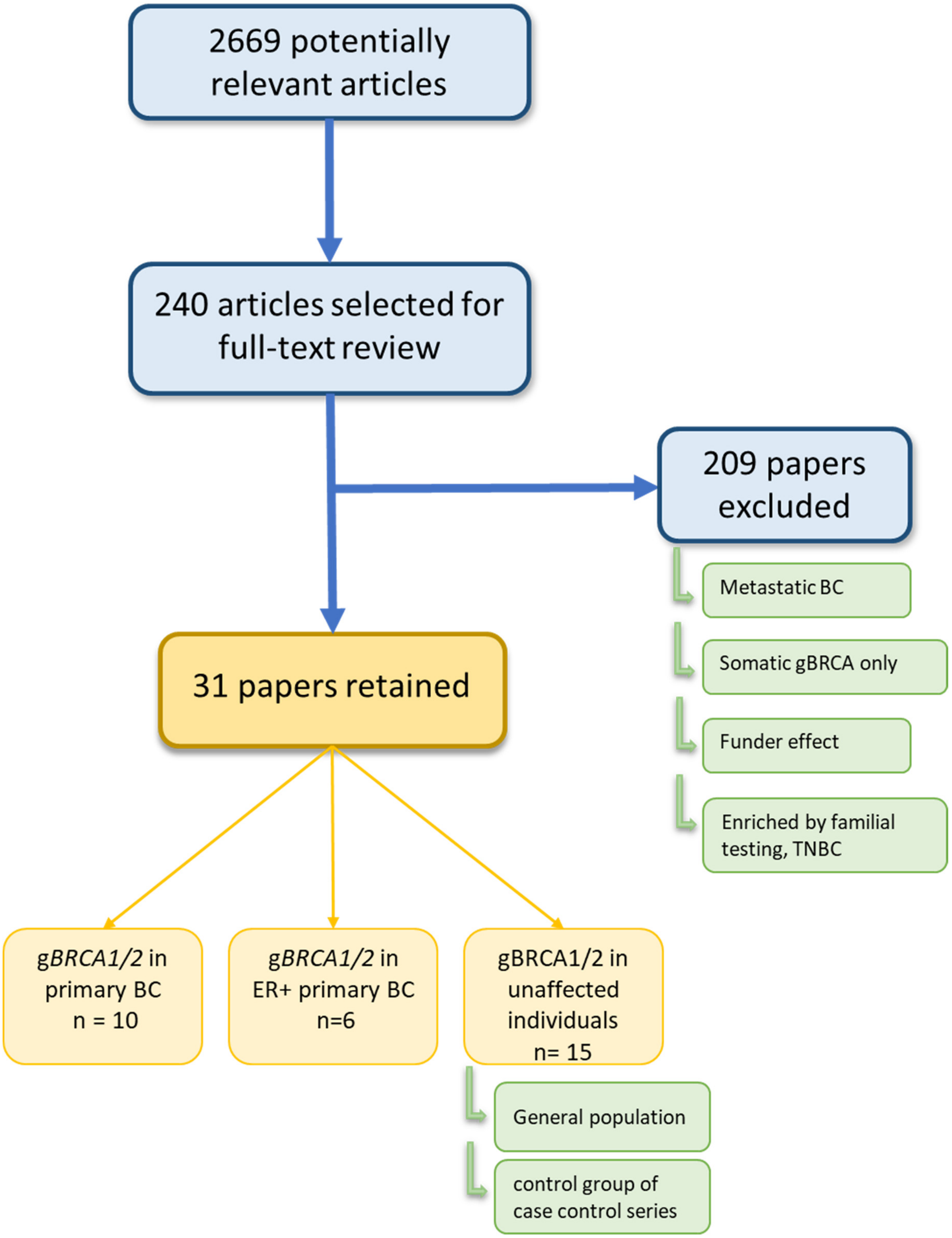

BC, breast cancer

Table S1: Frequency of gBRCA1 and gBRCA2 carriers among unselected breast cancer patients

Study	Breast cancer n	BRCA1 n	BRCA2 n
**Tung 2016**	488	18	12
**Hu 2021**	32,247	275	417
**Chen 2020**	524	11	18
**Pereira 2016***	2433	30	33
**Beitsch 2019**	959	2	13
**Abugattas 2015**	266	11	2
**Li 2019**	5,099	50	42
**Malone 2006**	1,628	52	44
**Kurian 2019**	18,522	536	477
**Dorling 2021**	48,826	515	754
**Total**	**108,699**	**1,496 (46%)**	**1,776 (54%)**

* from tumor mutation study;

1. Tung, N.; Lin, N.U.; Kidd, J.; Allen, B.A.; Singh, N.; Wenstrup, R.J.; Hartman, A.-R.; Winer, E.P.; Garber, J.E. Frequency of Germline Mutations in 25 Cancer Susceptibility Genes in a Sequential Series of Patients With Breast Cancer. *J. Clin. Oncol.* **2016**, *34*, 1460–1468.

2. Hu, C.; Hart, S.N.; Gnanaolivu, R.; Huang, H.; Lee, K.Y.; Na, J.; Gao, C.; Lilyquist, J.; Yadav, S.; Boddicker, N.J.; et al. A Population-Based Study of Genes Previously Implicated in Breast Cancer. *N. Engl. J. Med.* **2021**, *384*, 440–451.

3. Chen, B.; Zhang, G.; Li, X.; Ren, C.; Wang, Y.; Li, K.; Mok, H.; Cao, L.; Wen, L.; Jia, M.; et al. Comparison of BRCA versus Non-BRCA Germline Mutations and Associated Somatic Mutation Profiles in Patients with Unselected Breast Cancer. *Aging* **2020**, *12*, 3140–3155.

4. Pereira, B.; Chin, S.-F.; Rueda, O.M.; Vollan, H.-K.M.; Provenzano, E.; Bardwell, H.A.; Pugh, M.; Jones, L.; Russell, R.; Sammut, S.-J.; et al. The Somatic Mutation Profiles of 2,433 Breast Cancers Refines Their Genomic and Transcriptomic Landscapes. *Nat. Commun.* **2016**, *7*, 11479.

5. Beitsch, P.D.; Whitworth, P.W.; Hughes, K.; Patel, R.; Rosen, B.; Compagnoni, G.; Baron, P.; Simmons, R.; Smith, L.A.; Grady, I.; et al. Underdiagnosis of Hereditary Breast Cancer: Are Genetic Testing Guidelines a Tool or an Obstacle? *J. Clin. Oncol.* **2019**, *37*, 453–460.

6. Abugattas, J.; Llacuachaqui, M.; Allende, Y.S.; Velásquez, A.A.; Velarde, R.; Cotrina, J.; Garcés, M.; León, M.; Calderón, G.; de la Cruz, M.; et al. Prevalence of BRCA1 and BRCA2 Mutations in Unselected Breast Cancer Patients from Peru. *Clin. Genet.* **2015**, *88*, 371–375.

7. Li, J.; Wen, W.X.; Eklund, M.; Kvist, A.; Eriksson, M.; Christensen, H.N.; Torstensson, A.; Bajalica-Lagercrantz, S.; Dunning, A.M.; Decker, B.; et al. Prevalence of BRCA1 and BRCA2

Pathogenic Variants in a Large, Unselected Breast Cancer Cohort. *Int. J. Cancer* **2019**, *144*, 1195–1204.

8. Malone, K.E.; Daling, J.R.; Doody, D.R.; Hsu, L.; Bernstein, L.; Coates, R.J.; Marchbanks, P.A.; Simon, M.S.; McDonald, J.A.; Norman, S.A.; et al. Prevalence and Predictors of BRCA1 and BRCA2 Mutations in a Population-Based Study of Breast Cancer in White and Black American Women Ages 35 to 64 Years. *Cancer Res.* **2006**, *66*, 8297–8308.

9. Kurian, A.W.; Ward, K.C.; Howlader, N.; Deapen, D.; Hamilton, A.S.; Mariotto, A.; Miller, D.; Penberthy, L.S.; Katz, S.J. Genetic Testing and Results in a Population-Based Cohort of Breast Cancer Patients and Ovarian Cancer Patients. *J. Clin. Oncol.* **2019**, *37*, 1305–1315.

10. Breast Cancer Association Consortium; Dorling, L.; Carvalho, S.; Allen, J.; González-Neira, A.; Luccarini, C.; Wahlström, C.; Pooley, K.A.; Parsons, M.T.; Fortuno, C.; et al. Breast Cancer Risk Genes - Association Analysis in More than 113,000 Women. *N. Engl. J. Med.* **2021**, *384*, 428–439.

Table S2. Frequency of gBRCA1 and gBRCA2 carriers by ER status among unselected breast cancer patients

Study	Country/ Ethnicities	ER+ *	BRCA1/ ER+ n	BRCA2/ ER+ n	ER-	BRCA1/ ER-	BRCA2/ ER-
**Tung 2016**	United States: Boston, MA / 7.8% Ashkenazi Jewish 81.4% non-Ashkenazi white	364	5	11	124	13	1
**Hu 2021**	United States: 4% Asian, 12.3% Non-Hispanic black, 3.2% Hispanic, 78.9% Non-Hispanic white, 1.7% Other	18,428	73	201	3805	114	82
**Chen 2020**	South China	363	4	15	139	7	1
**Li 2019**	Sweden : Stockholm	3637	17	30	643	30	7
**Kurian 2019**	United States: California and Georgia	11642	227	365	7060	309	112
**Dorling 2021**	16 European countries, 5 Asian countries, 3 American countries 2 Oceania countries	*30466*	120	446	7766	269	149
**TOTAL**		**66680**	**457**	**1085**	**20190**	**761**	**368**

* including ER+PR- and ER+HER2+++ tumors;

Table S3. Frequency of gBRCA carriers among unaffected individuals

Type of study	Type of analysis	n	BRCA1	BRCA2	Reference
**Genome-wide population**					
	exome	50,726	95 (36%)	172 (64%)	Manickam 2018
	exome	30,223	86 (39%)	131 (61%)	Abul-Husn 2020
**Bio bank data**					
	exome and whole genome	125,747	332 (43%)	447 (57%)	gnomAD Karczewski2020
**Case–control**					
	large panel	32,544	37(32%)	78 (68%)	Hu2020 (CARRIERS)
	large panel	50,703	58 (30%)	135 (70%)	Dorling 2021
**Total**		**238,973 (1/173)**	**550 (40%) (1/434)**	**828 (60%) (1/288)**	

*https://gnomad.broadinstitute.org;

Table S4. Frequency of gBRCA carriers in gnomAD* exome sequencing database**

	Count		BRCA ratio		Prevalence			BRCA type prevalence difference
	BRCA1	BRCA2	BRCA1	BRCA2	BRCA1/2	BRCA1	BRCA2	P value***
**Complete cohort (n=125,747)**								
	332	447	43%	57%	0.62%	0.26%	0.36%	2*10-5
**Femalecohort (n=57,786)**								
	152	219	41%	59%	0.64%	0.26%	0.38%	3*10-4
**Male cohort (n=67,21961)**								
	180	228	44%	56%	0.60%	0.26%	0.34%	1*10-2

*v2.1.12; **using ClinVar August 2021. This database contains 7268 constitutive samples of individuals with cancer (5.7% of total, 3332 males and 3936 females). ***µFisher's exact test

References

1. Landrum, M.J.; Lee, J.M.; Benson, M.; Brown, G.R.; Chao, C.; Chitipiralla, S.; Gu, B.; Hart, J.; Hoffman, D.; Jang, W.; et al. ClinVar: Improving Access to Variant Interpretations and Supporting Evidence. *Nucleic Acids Res.* **2018**, *46*, D1062–D1067.

2. Karczewski, K.J.; Francioli, L.C.; Tiao, G.; Cummings, B.B.; Alföldi, J.; Wang, Q.; Collins, R.L.; Laricchia, K.M.; Ganna, A.; Birnbaum, D.P.; et al. The Mutational Constraint Spectrum Quantified from Variation in 141,456 Humans. *Nature* **2020**, *581*, 434–443.

Table S5. Meta-analysis of gBRCA carriers among unselected breast cancer patients

Data

	Study Name	Events	Sample size	Event rate	Logitevent rate	Std Err
**1**	Tung 2016	30	488	0.061	-2.726	**0.188**
**2**	Hu 2021	692	32247	0.021	-3.820	**0.038**
**3**	Chen 2020	29	524	0.055	-2.837	**0.191**
**4**	Pereira 2016	63	2433	0.026	-3.628	**0.128**
**5**	Beitsch 2019	15	959	0.016	-4.142	**0.260**
**6**	Abugattas 2015	13	266	0.049	-2.968	**0.284**
**7**	Li 2019	92	5099	0.018	-3.997	**0.105**
**8**	Malone 2006	96	1628	0.059	-2.770	**0.105**
**9**	Kurian 2019	1013	18522	0.055	-2.850	**0.032**
**10**	Dorling 2021	1269	48826	0.026	-3.624	**0.028**

Results

Study name	Event rate and 95% CI	Event rate	Lower limit	Upper limit	Z-Value
Tung 2016		0.061	0.043	0.087	-14.463
Hu 2021		0.021	0.020	0.023	-99.402
Chen 2020		0.055	0.039	0.079	-14.850
Pereira 2016		0.026	0.020	0.033	-28.417
Beitsch 2019		0.016	0.009	0.026	-15.916
Abugattas 2015		0.049	0.029	0.082	-10.438
Li 2019		0.018	0.015	0.022	-37.989
Malone 2006		0.059	0.049	0.072	-26.328
Kurian 2019		0.055	0.052	0.058	-88.187
Dorling 2021		0.026	0.025	0.027	-127.399
Total		0.034	0.025	0.047	-20.264

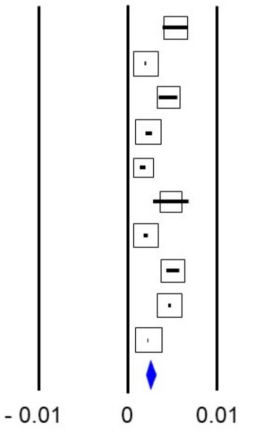

<u>Heterogeneity analysis</u>:
I2 statistic (%)= 94.7, Phet =0.000 (I2>40% p<0.05) ➔ heterogeneity ➔ random-effects model
<u>Forest plot</u>: gBRCA=3.4%, 95% CI 2.5-4.7

Selection Bias

			Random	
N studies	Eggers Test	Duval &Tweedie (DT) Method	DT Event rate	DT event rate IC 95%
10	0.88	0	0.034	0.025 – 0.047

Funnel plot log gBRCA

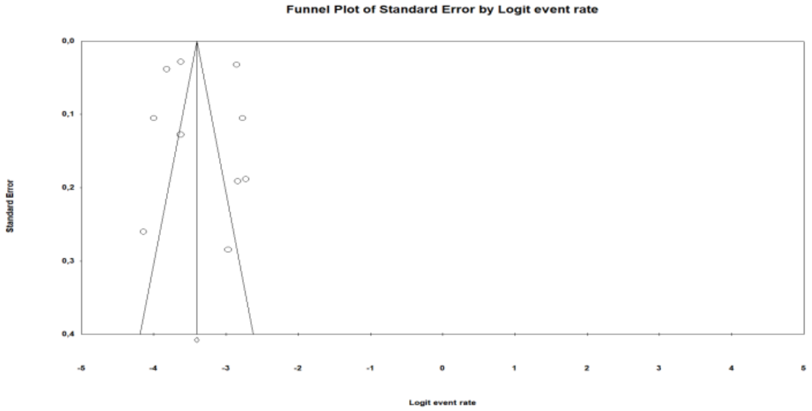

Results based on 10 studies retained (open circles). No additional studies identified by “trim and fill” (Egger et al, 1997; Duval & Tweedie, 2000). Egger’s test does not show publication bias (p=0.88).

Table S6. Meta-analysis of gBRCA carriers among ER+ breast cancer patients

Data

	Study Name	Events	Sample size	Event rate	Logitevent rate	Std Err
**1**	Tung 2016	16	364	0.044	-3.080	**0.256**
**2**	Hu 2021	274	18428	0.015	-4.194	**0.061**
**3**	Chen 2020	19	363	0.052	-2.896	**0.236**
**4**	Pereira 2016	28	1780	0.016	-4.136	**0.190**
**5**	Li 2019	47	3637	0.013	-4.336	**0.147**
**6**	Kurian 2019	592	11642	0.051	-2.927	**0.042**
**7**	Dorling 2021	566	30466	0.019	-3.967	**0.042**

Results

Study name — Event rate and 95% CI — Statistics for each study

Study name	Event rate	Lower limit	Upper limit	Z-Value
Tung 2016	0.044	0.027	0.071	-12.045
Hu 2021	0.015	0.013	0.017	-68.897
Chen 2020	0.052	0.034	0.081	-12.897
Pereira 2016	0.016	0.011	0.023	-21.714
Li 2019	0.013	0.010	0.017	-29.532
Kurian 2019	0.051	0.047	0.055	-69.375
Dorling 2021	0.019	0.017	0.020	-93.498
Total	0.025	0.015	0.041	-14.004

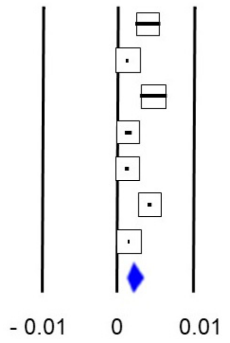

Heterogeneity analysis:
I^2 statistic (%)= 98.7, P_{het} =0.000 (I^2>40% p<0.05) ➔ heterogeneity ➔ random-effects model
Forest plot:
gBRCA=2.5%, 95% CI 1.5-4.1

Selection Bias

			Adjusted value : Random	
N studies	Eggers Test	Duval &Tweedie (DT) Method	DT Event rate	DT event rate IC 95%
7	0.77	1	0.022	0.014 – 0.036

Funnel plot log gBRCA

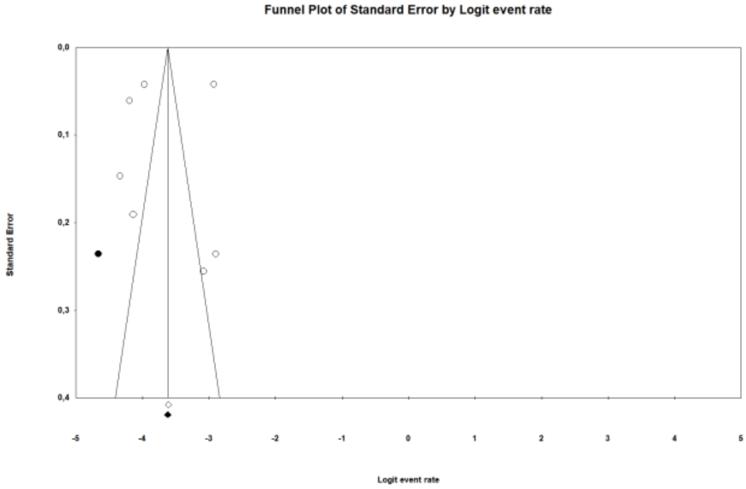

Results based on 7 studies retained (open circles). 1 additional study identified by "trim and fill" analysis (Egger et al, 1997; Duval & Tweedie, 2000). Egger's test does not show publication bias (p=0.77).

Table S7. Meta-analysis of gBRCA carriers among ER- breast cancer patients

Data

	Study Name	Events	Sample size	Event rate	Logitevent rate	Std Err
**1**	Tung 2016	14	124	0.113	-2.061	**0.284**
**2**	Hu 2021	196	3805	0.052	-2.913	**0.073**
**3**	Chen 2020	8	139	0.058	-2.796	**0.364**
**4**	Pereira 2016	35	653	0.054	-2.871	**0.174**
**5**	Li 2019	37	643	0.058	-2.796	**0.169**
**6**	Kurian 2019	421	7060	0.060	-2.758	**0.050**
**7**	Dorling 2021	418	7766	0.054	-2.867	**0.050**

Results

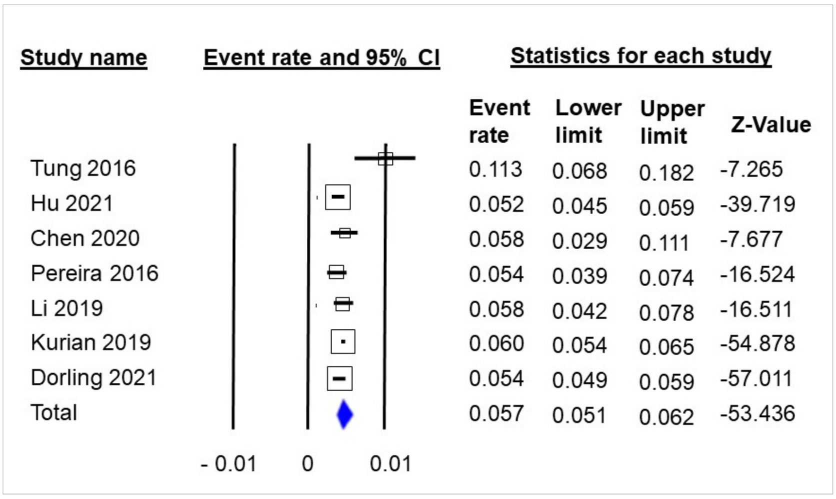

Heterogeneity analysis:
I^2 statistic (%)= 46.6, P_{het} =0.0810 ➔ heterogeneity ➔ random-effects model
Forest plot:
gBRCA=5.7%, 95% CI 5.1-6.2

Selection Bias

			Adjusted value : Random	
N studies	Eggers Test	Duval &Tweedie (DT) Method	DT Event rate	DT event rate IC 95%
7	0.43	0	0.057	0.051 – 0.062

Funnel plot log gBRCA

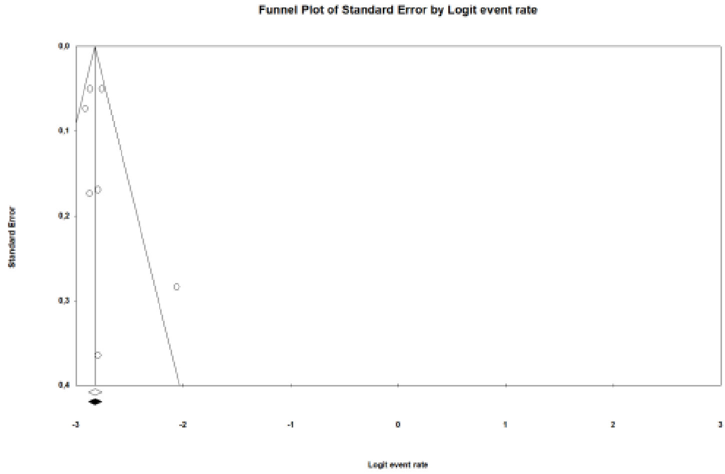

Results based on 7 studies retained (open circles). 1 additional study identified by “trim and fill” analysis (Egger et al, 1997; Duval & Tweedie, 2000). Egger’s test does not show publication bias (p=0.43).

Table S8. Meta-analysis of gBRCA carriers among unaffected individuals

Data

	Study Name	Events	Sample size	Event rate	Logitevent rate	Std Err
**1**	Genome-wide population / Manickam	267	50726	0.005	-5.242	**0.061**
**2**	Genome-wide population / Abul-Husn	217	30223	0.007	-4.929	**0.068**
**3**	Bio bank data / Karczewski 2020	779	125747	0.006	-5.078	**0.036**
**4**	Case-control large panel / Hu 2021	115	32544	0.004	-5.642	**0.093**
**5**	Case-control large panel / Dorling 2021	193	50703	0.004	-5.567	**0.072**

Results

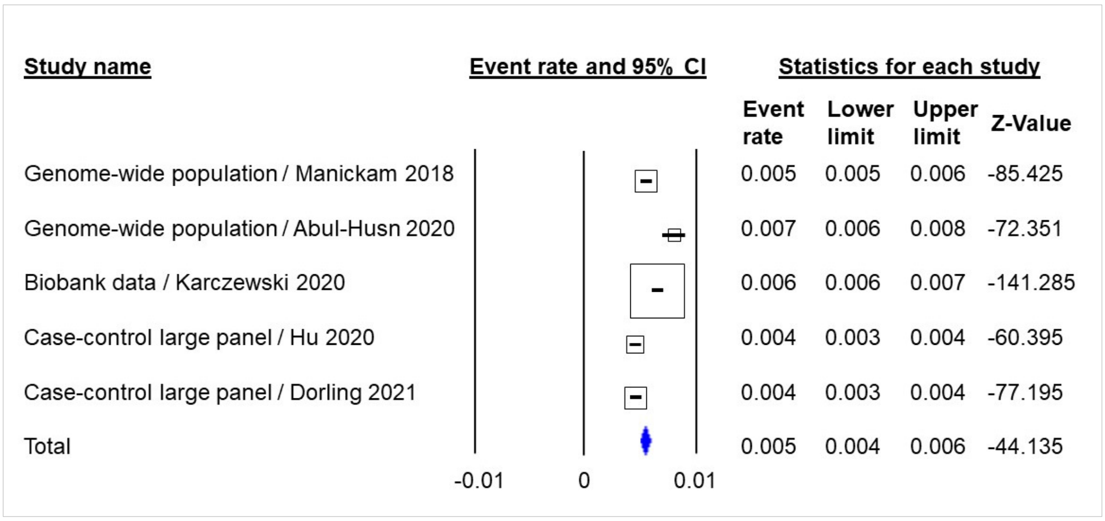

<u>Heterogeneity analysis</u>:
I^2 statistic (%)= 94.7, P_{het} =0.000 ➔ heterogeneity ➔ random-effects model
<u>Forest plot</u>:
gBRCA=0.5%, 95% CI 0.4-0.6

Selection Bias

			Adjusted value : Random	
N studies	Eggers Test	Duval &Tweedie (DT) Method	DT Event rate	DT event rate IC 95%
5	0.23	0	0.005	0.004 – 0.006

Funnel plot log gBRCA

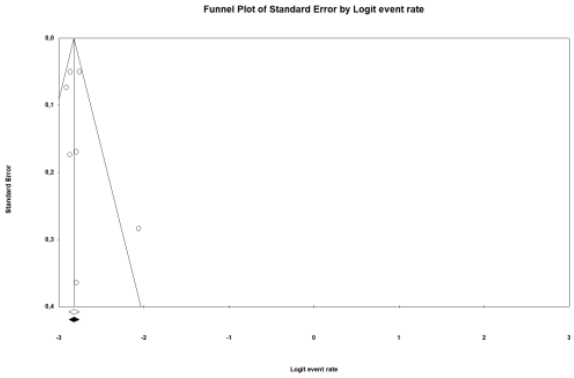

Results based on 5 studies or databases retained (open circles). No additional study identified by “trim and fill” analysis (Egger et al, 1997; Duval & Tweedie, 2000). Egger’s test does not show publication bias (p=0.23).

Table S9. Meta-analysis of gBRCA1 carriers among unselected breast cancer patients

Data

	Study Name	Events	Sample size	Event rate	Logitevent rate	Std Err
**1**	Tung 2016	18	488	0.037	-3.262	**0.240**
**2**	Hu 2021	275	32247	0.009	-4.756	**0.061**
**3**	Chen 2020	11	524	0.021	-3.842	**0.305**
**4**	Pereira 2016	30	2433	0.012	-4.383	**0.184**
**5**	Beitsch 2019	2	959	0.002	-6.171	**0.708**
**6**	Abugattas 2015	11	266	0.041	-3.143	**0.308**
**7**	Li 2019	50	5099	0.010	-4.615	**0.142**
**8**	Malone 2006	52	1628	0.032	-3.411	**0.141**
**9**	Kurian 2019	536	18522	0.029	-3.513	**0.044**
**10**	Dorling 2021	515	48826	0.011	-4.541	**0.044**

Results

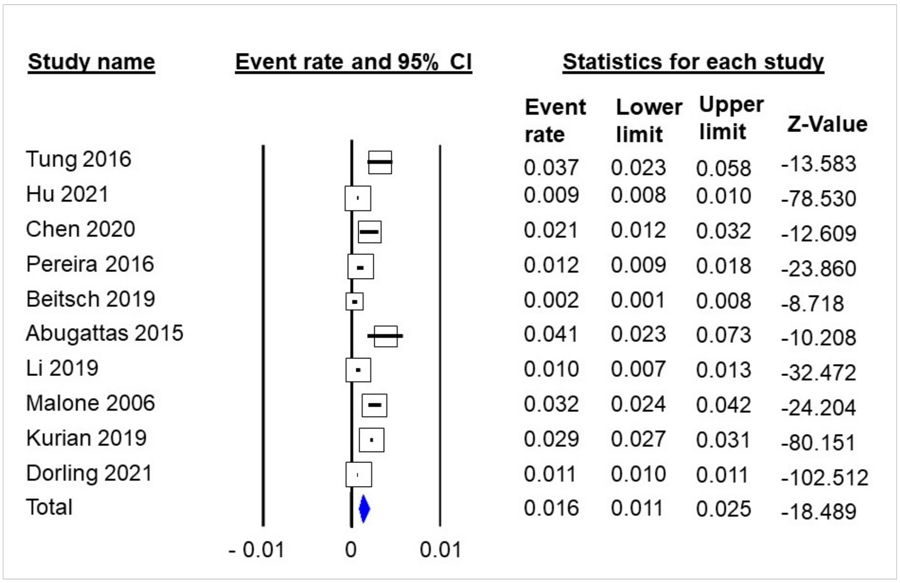

<u>Heterogeneity analysis</u>:

I^2 statistic (%)= 98, P_{het}0.000 (I^2>40% p<0.05) ➔ heterogeneity ➔ random-effects model

<u>Forest plot</u>:

gBRCA1=1.66%, 95% CI 1.08-2.54

Selection Bias

			Random	
N studies	Eggers Test	Duval &Tweedie (DT) Method	DT Event rate	DT event rate IC 95%
10	0.92	0	0.0166	0.0108 – 0.0254

Funnel plot log gBRCA1

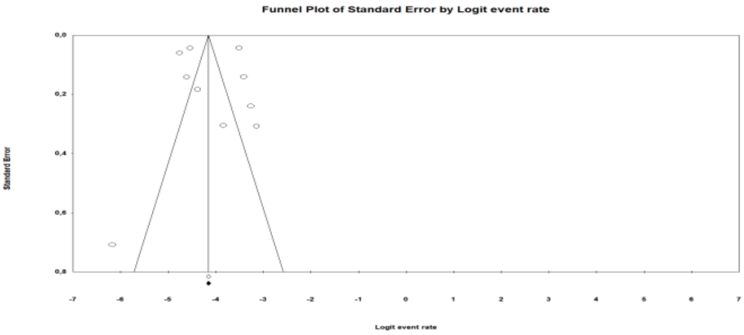

Results based on 10 studies retained (open circles). No additional study identified by "trim and fill" analysis (Egger et al, 1997; Duval & Tweedie, 2000). Egger's test does not show publication bias (p=0.92).

Table S10. Meta-analysis of gBRCA2 carriers among unselected breast cancer patients

Data

	Study Name	Events	Sample size	Event rate	Logitevent rate	Std Err
**1**	Tung 2016	12	488	0.025	-3.681	**0.292**
**2**	Hu 2021	417	32247	0.013	-4.335	**0.049**
**3**	Chen 2020	18	524	0.034	-3.336	**0.240**
**4**	Pereira 2016	33	2433	0.014	-4.287	**0.175**
**5**	Beitsch 2019	13	959	0.014	-4.287	**0.279**
**6**	Abugattas 2015	2	266	0.008	-4.883	**0.710**
**7**	Li 2019	42	5099	0.008	-4.791	**0.155**
**8**	Malone 2006	44	1628	0.027	-3.584	**0.153**
**9**	Kurian 2019	477	18522	0.026	-3.633	**0.046**
**10**	Dorling 2021	754	48826	0.015	-4.155	**0.037**

Results:

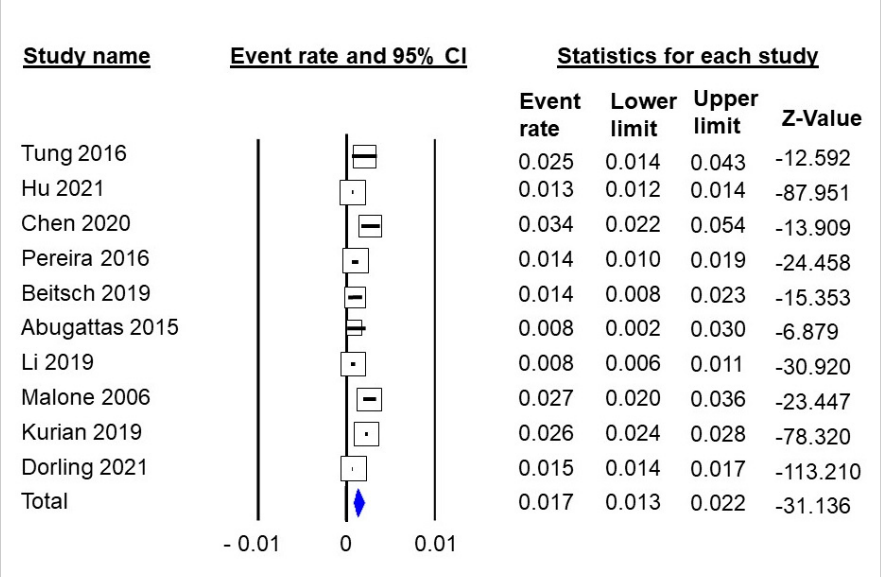

Heterogeneity analysis:

I^2 statistic (%)= 94.7, P_{het}=0.000 (I^2>40% p<0.05)) ➔ heterogeneity ➔ random-effects model

Forest plot:

gBRCA2=1.71%, 95% CI 1.33-2.2

Selection Bias

			Random	
N studies	Eggers Test	Duval &Tweedie (DT) Method	DT Event rate	DT event rate IC 95%
10	0.99	0	0.017	0.0133 – 0.022

Funnel plot log gBRCA2

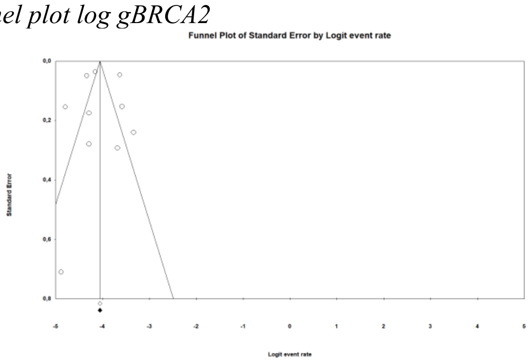

Results based on 10 studies retained (open circles). No additional study identified by "trim and fill" analysis (Egger et al, 1997; Duval & Tweedie, 2000). Egger's test does not show publication bias (p=0.99).

Table S11. Meta-analysis of gBRCA1 carriers among unaffected individuals

Data

	Study Name	Events	Sample size	Event rate	Logit event rate	Std Err
**1**	Genome-wide population exome 1	95	50726	0.002	-6.278	**0.103**
**2**	Genome-wide population exome 2	86	30223	0.003	-5.859	**0.108**
**3**	Bio bank data exome and whole genome	332	125747	0.003	-5.934	**0.055**
**4**	Case-control large panel 1	37	32544	0.001	-6.778	**0.164**
**5**	Case-control large panel 2	58	50703	0.001	-6.772	**0.131**

Results

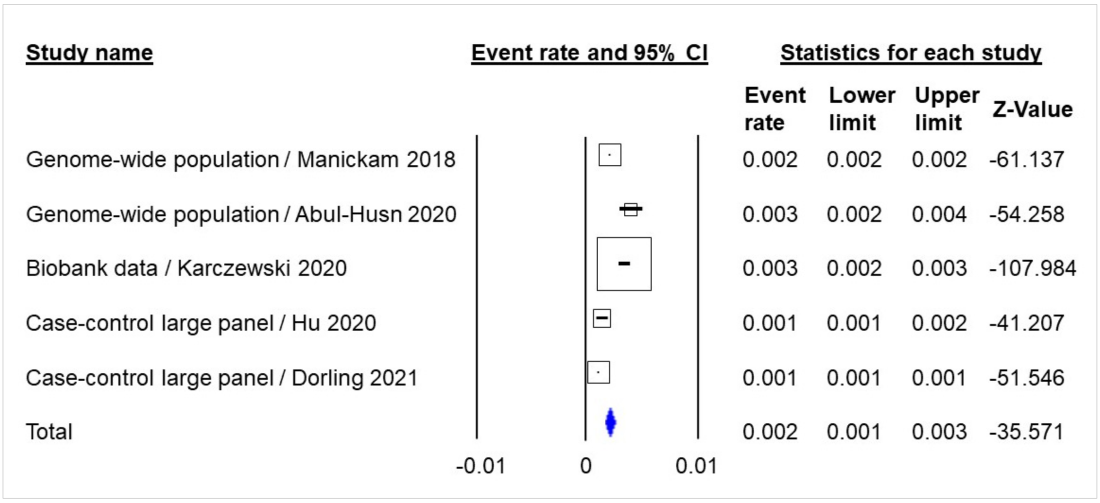

<u>Heterogeneity analysis</u>:
I^2 statistic =93%, p value 0.000 (I^2>40% p<0.05) ➔ heterogeneity ➔ random-effects model
<u>Forest plot</u>:
gBRCA1==0.182%, 95% CI 0.128-0.257

<u>Selection Bias</u>

			Random	
N studies	Eggers Test	Duval &Tweedie (DT) Method	DT Event rate	DT event rate IC 95%
5	0.22	0	0.00202	0.00147–0.00278

Funnel plot log gBRCA1

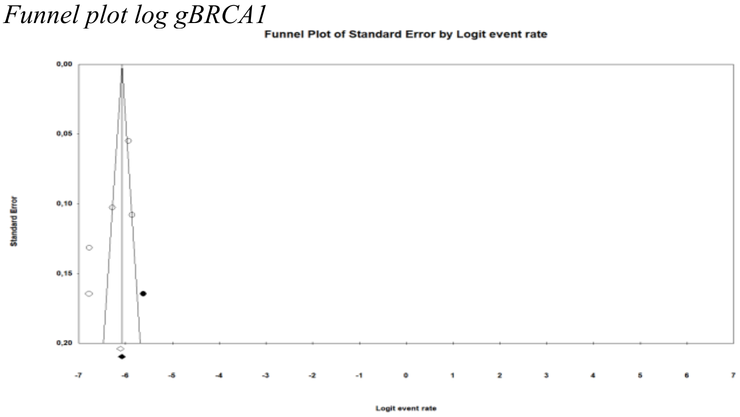

Results based on 5 studies or databases retained (open circles). 1 additional study identified by “trim and fill” analysis (Egger et al, 1997; Duval & Tweedie, 2000). Egger’s test does not show publication bias (p=0.22).

Table S12. Meta-analysis of gBRCA2 carriers among unaffected individuals

Data

	Study Name	Events	Sample size	Event rate	Logit event rate	Std Err
**1**	Genome-wide population exome 1	172	50726	0.003	-5.683	**0.076**
**2**	Genome-wide population exome 2	131	30223	0.004	-5.437	**0.088**
**3**	Bio bank data exome and whole genome	447	125747	0.004	-5.636	**0.047**
**4**	Case-control large panel 1	78	32544	0.002	-6.031	**0.113**
**5**	Case-control large panel 2	135	50703	0.003	-5.926	**0.086**

Results

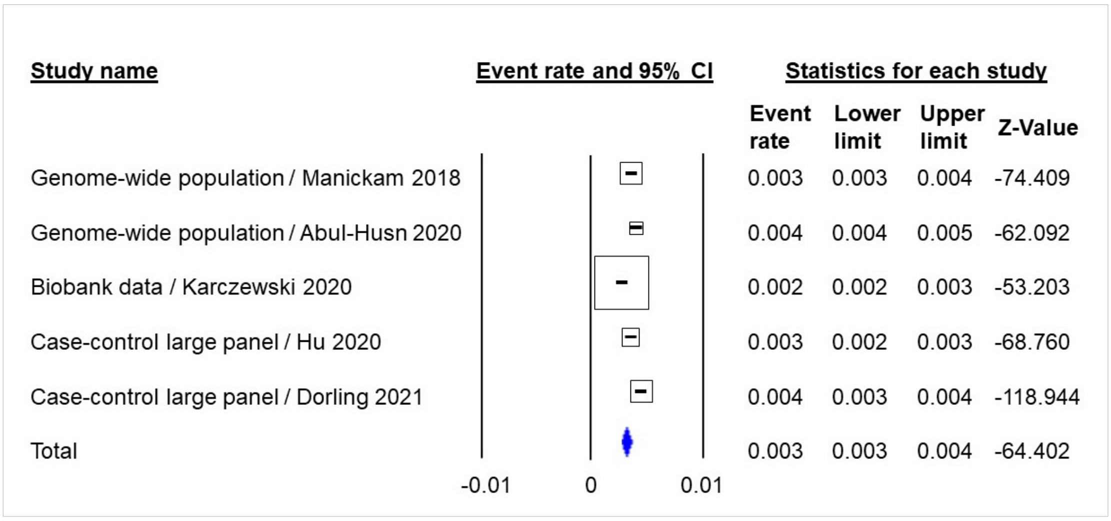

Heterogeneity analysis:
I^2 statistic =84.7%, p value 0.000 (I^2>40% p<0.05) ➔ heterogeneity ➔ random-effects model
Forest plot:
gBRCA2=0.323%, 95% CI 0.271-0.384

Selection Bias

			Random	
N studies	Eggers Test	Duval &Tweedie (DT) Method	DT Event rate	DT event rate IC 95%
5	0.42	0	0.00323	0.00271-0.00384

Funnel plot log gBRCA2

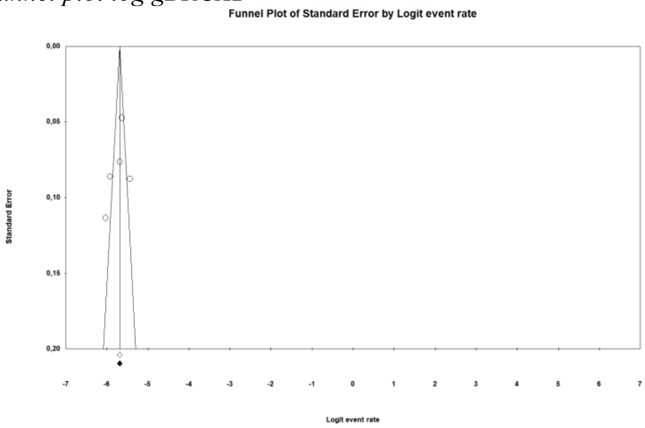

Results based on 5 studies or databases retained (open circles). No additional study identified by “trim and fill” analysis (Egger et al, 1997; Duval&Tweedie,2000).Egger test do not show publication bias publication bias (p=0.42).
